# Dr. Wollaston, the Chemist

**Published:** 1846-11

**Authors:** 


"*Dr. Wollaston, the Chemist.*—Littell's Living Age, that admirable digest of modern English literature, has a leading article, in the 125th No., extracted mainly from the British Quarterly Review, on the life, character, and discoveries of the late William Hyde Wollaston, M. D. Dr. W. will be known to posterity as a chemist, of extraordinary industry, patience and perseverance, whose discoveries have yielded important results to the arts and to the progress of various departments of science. Although acknowledged to be a renowned philosoper, he was a solitary being, as much as the last man will be, or as Adam was before the creation of Eve; and yet he resided in London. If society knew nothing of him, he certainly knew but little of society. His laboratory was so completely

*Andra..

a blue chamber, that only one person was ever in it besides himself, and that by mistake. The doctor discovered the intruder, and leading him to a particular part of the room, said, "Mr. P., do you see that furnace?" "I do." "Then make a profound bow to it, for as this is the first time, it will also be the last time of your seeing it." All his operations were conducted on a minute scale; a few grains of anything were enough, as a tray would hold all the apparatus and tests he seemed to require in his almost microscopic researches.

Dr. Wollaston was born Aug. 6, 1766, and died in December, 1828, having accumulated a handsome fortune by one single discovery, viz., a method of rendering platina malleable. For that one process, it is said that he realized 30,000*l.* When it is recollected that he invested 1000*l.* in the name of the Royal Society, the interest of which is to be employed to encourage experiments in natural philosophy, it hardly seems just to accuse him of being as money-loving as some have represented him. A single unlooked-for circumstance in the commencement of a professional life, sickened him of the practice of medicine, for which he was eminently well prepared by study, and although the people lost the services of a good physician, the whole world gained an illustrious philosopher. He was the second son of an eminent astronomer, and one of seventeen children. In 1783 he took the degree of M. D., and settled six weeks after, as a medical practitioner, at Bury St. Edmunds. So poor was his success, owing, beyond doubt, to the solemnity of his deportment, and never-relaxing reserve of manner, that he sought a larger field in London. That awful gravity which is forbidding in any one, and doubly so in a physician, who, of all men, should wear a cheerful face, drove patients away, or they were afraid to come; and he therefore solicited the office of physician to St. George's Hospital, a vacancy having occurred, recollecting, probably, that in such institutions, the sick are not at liberty to choose their medical advisers. To his chagrin, Dr. Pemberton obtained the appointment, which so mortified the learned chemist, who unquestionably felt conscious of possessing transcendent powers, that he declared he would abandon the profession, and never write another prescription, even for his own father. Having turned his attention afterwards wholly to chemical pursuits, the masterly efforts of a great mind were dicoverable in almost every inquiry to which his attention was directed. In the transactions of the Royal Society, principally, are to be found, from year to year, the details of his indefatigable labors. If he had not been decidedly a chemist, he would have been distinguished as an astronomer, since there was always a strong taste manifested for that elevated branch of knowledge. To particularize the names of the subjects on which he wrote, would be out of place in this brief notice of one who had the good fortune to escape the turmoils and competition, as well as the responsibilities, of a medical practitioner, and by doing so secured a conspicuous niche in the temple of fame.

The object in presenting this synopsis of the life of Dr. Wollaston, is to demonstrate the fact, that those who are as unsuccessful as he was in attempting to pursue the calling of a physician, are not to conclude they are equally unfit for every other pursuit, since they may rise much higher and perhaps be infinitely more useful, in some other way. Unceasing activity is required in those who hope for distinction in medicine and surgery, which distinction, when attained, is hardly a compensation for the toil it has cost Without the faculty of adapting oneself to society, as it exists, and the further ability to apply the knowledge that has been acquired according to the wants and necessities of the sick, success is a hopeless undertaking. Some men are constitutionally qualified for practising physic. Whether learned or ignorant, they succeed well. While others, like Dr. Wollaston, are incapable of success, but, without his firmness in resolve, cling with expectation to the wreck, fancying that success must ultimately crown their efforts—and there they remain, monuments of disappointed ambition."—*Boston Med. and Surg. Journal.*

